# Targeting both Notch and ErbB-2 signalling pathways is required for prevention of ErbB-2-positive breast tumour recurrence

**DOI:** 10.1038/bjc.2011.321

**Published:** 2011-08-16

**Authors:** K Pandya, K Meeke, A G Clementz, A Rogowski, J Roberts, L Miele, K S Albain, C Osipo

**Affiliations:** 1 Molecular Biology Program, Loyola University Chicago, 2160 South First Avenue, Maywood, IL 60153, USA; 2 Oncology Institute, Stritch School of Medicine at Loyola University Medical Center, 2160 South First Avenue, Maywood, IL 60153, USA; 3 Molecular and Cellular Biochemistry Program, Loyola University Chicago, 2160 South First Avenue, Maywood, IL 60153, USA; 4 University of Mississippi Cancer Institute, 350 Woodrow Wilson Drive, Suite 600, Jackson, MS 39213, USA; 5 Department of Medicine, Loyola University Chicago, 2160 South First Avenue, Maywood, IL 60153, USA; 6 Department of Pathology, 2160 South First Avenue, Maywood, IL 60153, USA; 7 Department of Microbiology and Immunology, 2160 South First Avenue, Maywood, IL 60153, USA

**Keywords:** ErbB-2, trastuzumab, Notch-1, GSI, recurrence, resistance

## Abstract

**Background:**

We reported that Notch-1, a potent breast oncogene, is activated in response to trastuzumab and contributes to trastuzumab resistance *in vitro*. We sought to determine the preclinical benefit of combining a Notch inhibitor (*γ*-secretase inhibitor (GSI)) and trastuzumab in both trastuzumab-sensitive and trastuzumab-resistant, ErbB-2-positive, BT474 breast tumours *in vivo*. We also studied if the combination therapy of lapatinib plus GSI can induce tumour regression of ErbB-2-positive breast cancer.

**Methods:**

We generated orthotopic breast tumour xenografts from trastuzumab- or lapatinib-sensitive and trastuzumab-resistant BT474 cells. We investigated the antitumour activities of two distinct GSIs, LY 411 575 and MRK-003, *in vivo.*

**Results:**

Our findings showed that combining trastuzumab plus a GSI completely prevented (MRK-003 GSI) or significantly reduced (LY 411 575 GSI) breast tumour recurrence post-trastuzumab treatment in sensitive tumours. Moreover, combining lapatinib plus MRK-003 GSI showed significant reduction of tumour growth. Furthermore, a GSI partially reversed trastuzumab resistance in resistant tumours.

**Conclusion:**

Our data suggest that a combined inhibition of Notch and ErbB-2 signalling pathways could decrease recurrence rates for ErbB-2-positive breast tumours and may be beneficial in the treatment of recurrent trastuzumab-resistant disease.

We have recently shown that the epidermal growth factor receptor-2 (ErbB-2 or HER-2/neu) inhibits Notch-1 activity (Osipo *et al*, 2008). The ErbB-2 is a receptor tyrosine kinase that belongs to the epidermal growth factor receptor family. It is activated by growth factor-independent homodimerisation or growth factor-dependent heterodimerisation with family members, epidermal growth factor receptor (EGFR), ErbB-3, or ErbB-4 (Yarden, 2001). The ErbB-2 transmits its signal inside the cell to stimulate proliferation and survival (Yarden, 2001; Yarden and Sliwkowski, 2001). The ErbB-2 proto-oncogene is amplified, overexpressed, and/or hyperactive in 15–25% of patients with breast cancers, such as the ‘HER-2 positive subtype’, which is oestrogen receptor *α* (ER*α*) and progesterone receptor (PR) negative, or a subset of the ‘luminal B subtype’, which is ER*α*/PR positive (Slamon *et al*, 1989; Menendez *et al*, 2006). The clinical importance of targeted therapy based on increased expression and activity of ErbB-2 is underestimated if just based on the ER*α*/PR classification. This is because it has also been shown that the luminal A (ER*α*/PR+, HER-2−) subtype of breast tumours, which represents ∼70% of breast cancers, might, after becoming resistant to antihormonal therapy such as tamoxifen (Benz *et al*, 1992; Osipo *et al*, 2003, 2005a) or aromatase inhibitors (Brodie *et al*, 2009), evolve to overexpress ErbB-2. Some luminal B and the HER-2+ subtypes of breast cancer, which contain gene amplification of ErbB-2, are associated with poor prognosis, aggressive disease, and resistance to some cytotoxic (Yokoyama *et al*, 2005) and endocrine therapies (Shou *et al*, 2004). Furthermore, the increased ErbB-2 activity in the luminal A tumours that become resistant to antihormonal therapy may be an important target for alternative or combination therapy that attempts to prevent and/or reverse the resistance to antihormonal therapy.

Trastuzumab is a humanised monoclonal antibody that binds the extracellular, juxtamembrane domain of ErbB-2 (Carter *et al*, 1992). Trastuzumab blocks ErbB-2 at the cell surface and inhibits receptor–receptor interactions, thereby slowing growth by inhibiting activation and signalling (Sliwkowski *et al*, 1999; Baselga *et al*, 2001; Nagata *et al*, 2004; Nahta and Esteva, 2006). The best efficacy and positive therapeutic outcome with trastuzumab is observed in women with tumours that overexpress, have an amplification, or have high activity of ErbB-2 (Muss *et al*, 1994; Nabholtz and Slamon, 2001; Slamon and Pegram, 2001; Menendez *et al*, 2006). Although trastuzumab has had a tremendous impact on improving survival for women with ErbB-2-positive breast cancer, resistance to trastuzumab remains a serious and unacceptable clinical problem, particularly in women with metastatic breast cancer. Unfortunately, when given as a single agent, 66–88% of women with metastatic breast cancer are resistant to trastuzumab (Cobleigh *et al*, 1999; Vogel *et al*, 2002). Furthermore, some women with metastatic breast cancer, who initially respond to trastuzumab-based treatments that include chemotherapy, develop resistance within the first year (Amar *et al*, 2008). Approximately 15% of women with non-metastatic breast tumours who receive trastuzumab in the adjuvant setting will develop recurrent breast cancer. Recently, lapatinib, a small-molecule, dual EGFR/ErbB-2 tyrosine kinase inhibitor (TKI), has been effective in women with trastuzumab-resistant disease when combined with a cytotoxic agent, the selective oral fluoropyrimidine capecitabine that is ultimately converted to 5-fluorouracil (Nahta *et al*, 2007). However, lapatinib resistance has been observed in cell culture models (Xia *et al*, 2006) and, clinically, patients treated with lapatinib become resistant within the first year of treatment (Li *et al*, 2008; Widakowich *et al*, 2008; Kaufman *et al*, 2009; Lacerda *et al*, 2010). Thus, despite initial efficacy in the treatment of metastatic disease with agents that target the ErbB receptor pathways, resistance occurs with few clinical means currently available to circumvent it (Li *et al*, 2008).

We have identified Notch-1 as a novel target in trastuzumab-resistant breast cancer (Osipo *et al*, 2008). Notch-1 is a type I membrane receptor that is a breast oncogene (Dievart *et al*, 1999) and a potent cell-fate modulator (Politi *et al*, 2004). Increased coexpression of Notch-1 and its ligand Jagged-1 predicts the poorest overall survival in women with breast cancer (Reedijk *et al*, 2005; Dickson *et al*, 2007). Furthermore, Notch-1 mRNA was found to be elevated in breast cancer cells resistant to gefitinib, an EGFR TKI (Piechocki *et al*, 2007). There are four mammalian Notch receptors (Notch-1, -2, -3, and -4) with five canonical ligands (Delta-like 1, 3, and 4 (Bettenhausen *et al*, 1995; Dunwoodie *et al*, 1997; Shutter *et al*, 2000) and Jagged-1 and -2 (Lindsell *et al*, 1995)). Both Notch ligands and receptors are type I membrane proteins, and Notch receptor–ligand interactions regulate cell fate and have been shown to be important for survival of breast tumour-initiating cells (Magnifico *et al*, 2009; Mine *et al*, 2009; Harrison *et al*, 2010). Notch receptors have two subunits, an extracellular Notch (N^EC^) and a transmembrane Notch (N^TM^) (Blaumueller *et al*, 1997; Logeat *et al*, 1998). Dissociation of N^EC^ and N^TM^ subunits is initiated upon ligand binding, which triggers two proteolytic cleavages of N^TM^. The final cleavage within the Notch transmembrane domain is catalysed by *γ*-secretase, which releases the C-terminal, intracellular portion of the Notch receptor (NIC). This final step can be pharmacologically inhibited by *γ*-secretase inhibitors (GSIs), which prevent the release of NIC, thus inhibiting Notch-mediated transcription and cell growth. The GSIs are currently in clinical trials for the treatment of breast cancer and other solid tumours (Pannuti *et al*, 2010). The NIC modulates transcription via the CSL (CBF-1) transcription factor in the nucleus. Notch signalling contributes to breast cancer tumourigenesis by inhibiting differentiation, promoting survival, and/or accelerating proliferation. Here, we used three preclinical xenograft models *in vivo*, trastuzumab-sensitive, trastuzumab-resistant, and lapatinib-sensitive xenografts, to investigate the anti-tumour benefit of combining trastuzumab or lapatinib plus a GSI on the growth of ErbB-2-positive breast tumours.

## Materials and methods

### Development of BT474 trastuzumab-sensitive, trastuzumab-resistant, and lapatinib-sensitive xenografts

BT474 cells were purchased from the American Tissue Cell Culture (ATCC, Manassas, VA, USA) and maintained as described previously (Osipo *et al*, 2008). BT474 breast cancer cells that are sensitive or resistant to trastuzumab as described previously (Osipo *et al*, 2008) and sensitive to lapatinib were used to generate breast tumour xenografts. Five million cells were injected into two mammary fat pads of ovariectomised, FoxN1^nu/nu^ athymic nude mice (Harlan Sprague-Dawley, Madison, WI, USA) followed by implantation of a 17*β*-estradiol-containing silastic capsule of 0.3 cm in length with a constant release providing 83–100 pg ml^−1^ as described previously (O’Regan *et al*, 1998). Each mouse was tagged on one ear to identify the specific mouse and tumour. Once tumours grew to a mean cross-sectional area (CRA) of 0.20–0.30 cm^2^, mice were killed; tumours were extracted and re-transplanted into a set of 56 mice as previously described (Osipo *et al*, 2005b). Tumours were allowed to grow to a mean CRA of 0.20–0.30 cm^2^ and mice were randomised to four treatment groups with 14 mice per group: vehicle control (sterile PBS and 2% carboxymethylcellulose), trastuzumab (10 mg kg^−1^ in a total volume of 100 *μ*l sterile PBS, i.p. once a week) or lapatinib (Selleck Chemical, Houston, TX, USA; 100 mg kg^−1^; fed by oral gavage, twice daily for 5 days), LY 411 575 GSI (kindly provided by Drs Abdul Fauq and Todd Golde from the Mayo Clinic in Jacksonville, FL, USA; 5 mg kg^−1^; 3 days on, 4 days off) or MRK-003 (kindly provided by Merck & Co., Whitehouse Station, NJ, USA; 100 mg kg^−1^ dissolved in 2% carboxymethylcellulose, 200 *μ*l fed by oral gavage; 3 days on, 4 days off), or trastuzumab plus MRK-003 or LY 411 575 GSI or lapatinib plus MRK-003 GSI. Tumour area (l × w) was measured weekly using Vernier calipers and cross-sectional area ((l × w)Π)/4) was calculated and graphed. Tumour recurrence was monitored and tumour area measured after treatments with trastuzumab or trastuzumab plus GSI were ceased at the time tumours were no longer detectable by the Vevo 770 Ultrasound Imaging system (Visual Sonics, Toronto, Canada). Tumour recurrence post treatment was monitored up to ∼100 days. Protocols that were used to study breast tumour xenografts in mice were approved by Loyola University's Institutional Animal Care and Use Committee.

### Immunohistochemistry

Tumour sections (4 *μ*m) were sliced from formalin-fixed, paraffin-embedded tumour samples, followed by antigen retrieval to unmask the epitope. Immunohistochemical staining was performed as described previously (Yao *et al*, 2010) using an antibody against the proliferation marker, Ki67 (proliferation marker, 1 : 100, DAKO, Carpinteria, CA, USA), for all four treatment groups. Biotinylated anti-mouse secondary antibody (VECTASTAIN Elite ABC Kit, Vector Laboratories, Burlingame, CA, USA) was applied to the slides to detect the primary antibody, followed by incubation with the avidin-horseradish peroxidase complex reagent (VECTASTAIN Elite ABC kit, Vector Laboratories). The staining was developed in the diaminobenzidine chromogen substrate solution (Peroxidase Substrate Kit, Vector Laboratories). Mayer's haematoxylin was used as a counterstain. Immunohistochemical analysis of Ki67 was performed using at least 3–5 tumours per treatment group. The number of Ki67-positive cells as shown on the *y* axis was determined by taking an average number of Ki67-positive cells from 60 high-power fields (HPFs) at × 40 magnification per treatment group. Haematoxylin and eosin (H/E) was performed to ensure the presence of tumour cells in the tumour sections.

### TUNEL assay

Terminal deoxynucleotidyl transferase-mediated dUTP nick end labelling (TUNEL) assay with the TMR detection kit (Roche Diagnostic, Indianapolis, IN, USA) was used to detect 3′OH-associated DNA fragmentation resulting from apoptosis. Paraffin-embedded BT474 trastuzumab- and lapatinib-sensitive tumours extracted from xenografts as described above were examined for the presence of TUNEL-positive cells from each treatment group according to the manufacturer's instructions. The TUNEL assay was performed using 3–5 tumours from each treatment group. The number of TUNEL-positive cells shown on the *y* axis was the average number of TUNEL-positive cells counted per 20 HPFs per tumour for a total of 60 HPFs at × 40 magnification per treatment group.

### Western blot analysis

Frozen tumour samples from each treatment group were homogenised by grinding in liquid nitrogen and lysed in lysis buffer (50 mM HEPES, 1% Triton X-100, 150 mM NaCl, 5 mM EDTA, 10 *μ*g ml^−1^ pepstatin A, 10 *μ*g ml^−1^ leupeptin, 10 *μ*g ml^−1^ aprotinin, 25 *μ*g ml^−1^ PMSF, 10 *μ*g ml^−1^ TLCK, 10 *μ*g ml^−1^ TPCK, 1 mM o-vanadate, and 10 mM NaF). The lysate was sonicated, followed by centrifugation at 10 000 g for 5 min. The supernatant was collected and protein concentration was measured using the BCA protein assay (Thermo Fisher Scientific, Inc., Waltham, MA, USA). Tumour lysates were then subjected to SDS–PAGE and western blotting (Osipo *et al*, 2008) was performed using the following antibodies: phosphorylated tyrosine (Y1248)-ErbB-2 (1 : 1000, Millipore, Billerica, MA, USA), total ErbB-2 (1 : 500, AB-17, Thermo Fisher Scientific), phosphorylated-ERK1/2 (1 : 1000, Cell Signaling Technology, Danvers, MA, USA), total ERK1/2 (1 : 1000, Cell Signaling Technology), phosphorylated-AKT1 (1 : 1000, Cell Signaling Technology), total AKT1 (1 : 300, Santa Cruz Biotechnology, Santa Cruz, CA, USA), PTEN (1 : 1000, Cell Signaling Technology), and Actin as a loading control (1 : 3000, Sigma-Aldrich, St Louis, MO, USA). Anti-mouse (1 : 3000, Santa Cruz Biotechnology) or anti-rabbit (1 : 2000, Santa Cruz Biotechnology) secondary antibodies conjugated to horseradish peroxidase were used to detect the primary antibody. The blot was then developed using SuperSignal West Dura Luminol/Enhancer Solution (Thermo Fisher Scientific, Inc.) and the stained bands were visualised using the Fujifilm LAS-3000 imager (Valhalla, NY, USA).

### Reverse transcription real-time PCR

Total RNA was extracted from frozen tumour samples using the RiboPure Kit (Ambion, Inc., Austin, TX, USA) according to the manufacturer's instructions. Total RNA was reverse transcribed to total cDNA using the TaqMan reverse transcription Kit (Applied Biosystems, Foster City, CA, USA) according to the manufacturer's instructions. Real-time PCR was performed using cDNA as template to detect relative expression levels of Notch targets such as *HEY1* (forward primer: 5′-AGCTCCTCGGACAGCGAGCTG-3′, reverse primer: 5′-TACCAGCCTTCTCAGCTCAGACA-3′) and *Deltex1* (forward primer: 5′-CAGTTTCGCCAGGACACAG-3′, reverse primer: 5′-GCAGATGTCCATATCGTAGGC-3′). The expression level of 18S (forward primer: 5′-ATGAACCAGGTTATGACCTTGAT-3′, reverse primer: 5′-CCTGTTGACTGGTCATTACA-ATA-3′) was used as a loading control. The PCR was performed as previously described (Osipo *et al*, 2008).

### Statistical analysis

Karyn Richlyk, a statistician at Loyola University Medical Center, assisted in determining the number of mice needed for the *in vivo* study. Based on experience, we hypothesised the following average tumour size for the four groups in trastuzumab- or lapatinib-sensitive xenograft studies at the end of the experiment (all measurements are in cross-sectional area=cm^2^): 1 vehicle=2.0 (s.d.=0.3); 2 trastuzumab or lapatinib=0.4 (s.d.=0.1); 3 GSI=1.5 (s.d.=0.1); and 4 GSI+trastuzumab or lapatinib <0.1 (s.d.=0.01). For the trastuzumab-resistant xenograft study, the average tumour size for vehicle, GSI, and GSI+trastuzumab should remain the same as above. However, as these are trastuzumab-resistant tumours, we would expect the average tumour size for the trastuzumab group as 1.5 cm^2^. Calculations were conducted using PASS 2002 software (Kaysville, UT, USA, 2002). In a one-way ANOVA, same sample sizes of 7 were obtained for all the four groups whose means are to be compared, assuming 100% tumour take. The total sample of 28 mice achieves 95% power to detect differences among the means *vs* the alternative of equal means using an F-test at a significance level of 0.05. The common s.d. within a group is assumed to be between 1 and 0.01. However, experience suggests that tumour take will be 50–70% therefore, in order to maximise the likelihood that 7 subjects per group will present with tumours, we must assume that a sample of 7 represents 50–70% from a group of 14 mice, for a total of 56 mice per experiment of four groups. Each mouse was identifiable with a numbered tag. Each tumour area on the left flank and right flank of the mouse was measured weekly with Vernier calipers. At the end of the study, tumour CRA was calculated and linear regression analysis was performed to determine the slope of the line for determination of the rate of growth for each tumour. Slopes of lines were used only if the correlation coefficients were ⩾0.85. A one-way ANOVA with Bonferroni correction for multiple comparisons and *α*=0.05 was used to test statistical significance between groups for tumour growth rates, mRNA expression levels, and IHC assays. A nonpaired Student's *t*-test was used to test statistical significance between two groups.

## Results

### Anti-tumour activity of trastuzumab, GSIs, or combinations in ErbB-2-positive breast tumours *in vivo*

**Figure 1:**
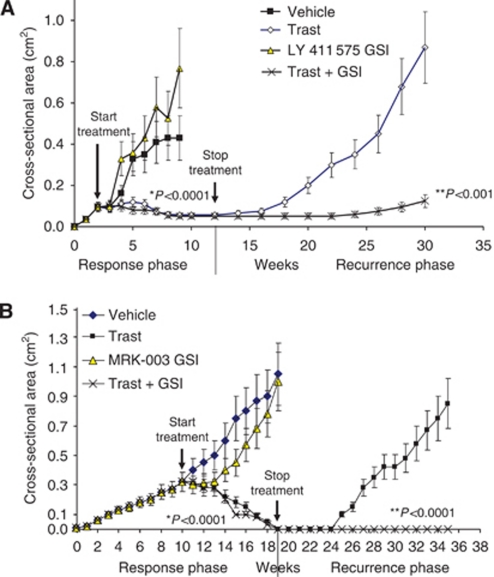
Trastuzumab (Trast) plus a *γ*-secretase inhibitor (GSI) prevents or reduces tumour recurrence. ErbB-2-overexpressing BT474 xenografts were generated in 56 ovariectomised, athymic nude mice by injecting 5 × 10^6^ cells into both mammary fat pads. Once tumours reached a mean tumour cross-sectional area of 0.20 cm^2^, mice were randomised and treated with vehicle (100 *μ*l sterile PBS injected i.p. 1 day per week and 200 *μ*l 2% carboxymethylcellulose), 10 mg kg^−1^ trastuzumab in 100 *μ*l PBS injected i.p. once weekly, 5 mg kg^−1^ LY 411 575 GSI (**A**) or 100 mg kg^−1^ MRK-003 GSI (**B**) in 200 *μ*l 2% carboxymethylcellulose, fed by oral gavage, three days on, 4 days off, or trastuzumab plus LY 411 575 GSI or MRK-003 GSI. Tumour area (length × width) was measured weekly using Vernier calipers. The measurements were performed up to 12 or 19 weeks. The treatments were stopped and tumour recurrence was measured up to an additional 105 days or 98 days in mice that specifically showed complete tumour regression (**A** and **B**). Results from (**A** and **B**) show mean tumour cross-sectional area ((area × Π)/4) on the *y* axis and time in weeks on the *x* axis. Error bars are s.d. of the mean for 12 mice bearing tumours in the response phase of the study and 8 mice for the recurrent phase of the study. The results from (**A** and **B**) also demonstrate mice bearing recurrent tumours on the *y* axis and treatments on the *x* axis. ^*^Statistically significant differences between mean slopes of the curve for trastuzumab plus GSI *vs* GSI alone. ^**^Statistically significant differences between mean slopes of the curve for trastuzumab *vs* trastuzumab plus GSI in recurrent tumours. Linear regression analyses were performed for tumour growth curves in (**A** and **B**).

We used several cell lines in our *in vitro* studies (Osipo *et al*, 2008); however, we chose to use BT474 ErbB-2-positive breast cancer cells as they are sensitive to trastuzumab and have been used numerous times in preclinical studies (Nagata *et al*, 2004; Rimawi *et al*, 2011) to represent the clinical setting. Osipo *et al* (2008) showed that ErbB-2 overexpression suppresses Notch-1 activity; thus, BT474 cells, which contain a gene amplification and therefore overexpress ErbB-2, exhibit minimal Notch-1 activity. Conversely, trastuzumab treatment increases Notch-1 transcriptional activity five-fold, and this effect was abrogated by using a GSI (Osipo *et al*, 2008). Breast tumour xenografts were generated using BT474 trastuzumab-sensitive cells in athymic nude mice. Growth of tumours was measured in response to vehicle, trastuzumab, LY 411 575 or MRK-003 GSI, or trastuzumab plus GSI. The results from two independent studies showed that trastuzumab treatment almost completely inhibited the tumour growth of BT474 breast tumour xenografts with ∼90–100% of tumours regressing to undetectable levels (Figure 1A and B). GSI treatment of tumours, either alone using LY 411 575 (Figure 1A) or MRK-003 (Figure 1B) or in combination with trastuzumab, had no statistically significant effect on tumour growth during the treatment phase of the study compared with vehicle control or trastuzumab alone, respectively.

**Figure 2:**
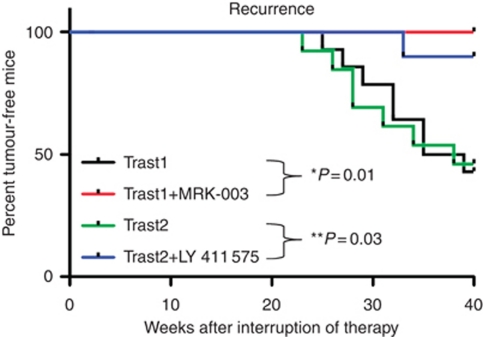
Kaplan–Meier curve of the percentage of tumour-free mice among Trast1, Trast1+MRK-003 GSI, Trast2, and Trast2+LY 411 575 GSI treatment groups using a log rank (Mantel–Cox) test. ^*^Statistically significant differences between Trast1 and Trast1 + MRK-003 GSI. ^**^Statistically significant differences between Trast2 and Trast2 + LY 411 575 GSI.

There is significant evidence of enhanced Notch signalling in tumour-initiating or putative breast cancer stem cells (Grudzien *et al*, 2010; Harrison *et al*, 2010; Pannuti *et al*, 2010). As these cells are suggested to be responsible for tumour recurrence, we discontinued treatment and measured tumour recurrence in mice where tumours were no longer detectable using the Vevo 770 Ultrasound Imager. During the recurrence phase of the study, we detected significant tumour re-growth for the previously trastuzumab-treated mice only (Figure 1A and B). In contrast, mice previously treated with the combination of trastuzumab plus LY 411 575 GSI did not display tumour re-growth until week 25, and their tumours were significantly smaller (0.12 *vs* 0.87 cm^2^ for the trastuzumab-alone group, *P*<0.001; Figure 1A). Furthermore, no recurrent tumours were detectable in mice previously treated with the combination of trastuzumab plus MRK-003 GSI (*P*<0.0001; Figure 1B). Figure 2 is a Kaplan–Meier analysis of log-rank (Mantel–Cox) test for the rate of tumour recurrence post treatment. In both studies, ∼40% of mice previously treated with trastuzumab alone displayed tumour recurrence at 40 weeks. In contrast, only 10% of mice displayed tumour recurrence in the trastuzumab plus LY 411 575 GSI group, and 0% of mice displayed tumour recurrence in the trastuzumab plus MRK-003 group (Figure 2). These data suggest that the main benefit of using a GSI (MRK-003) in trastuzumab-sensitive, ErbB-2-positive breast tumours is prevention of tumour recurrence. All mice were killed at the end of 40 weeks and it was confirmed via surgery that tumours were completely absent in the trastuzumab plus MRK-003 group.

### Expression of Notch gene targets: *HEY1* and *Deltex1*

**Figure 3:**
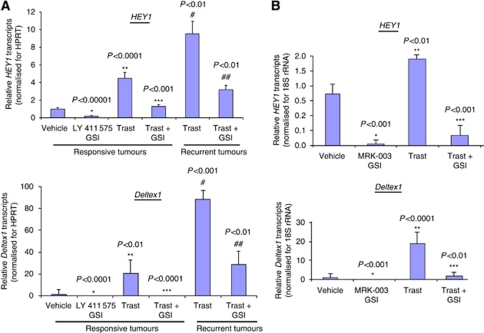
Activity of the Notch-1 signalling pathway in BT474 tumours. (**A** and **B**) In a separate experiment, 1 mg of snap-frozen tumours were homogenised and lysed in Trizol reagent and total RNA extracted using the Trizol protocol as described previously (Osipo *et al*, 2008). Total RNA was reverse transcribed to total cDNA using the Applied Biosystems kit. Real-time PCR was performed using human-specific primers to detect transcripts from Notch target genes: human *HEY1* and human *Deltex1*. Human-specific 18S rRNA was detected for normalisation. Results are mean relative transcripts levels compared with vehicle (Control) after normalisation to 18S rRNA. Error bars are s.d. of the mean for five independent tumour samples. ^*^Statistically significant differences between MRK-003 or LY 411 575 GSI and vehicle (Control). ^**^Statistically significant differences between trastuzumab (Trast) and Control. ^***^Statistically significant differences between GSI + Trast and Trast alone. ^#^Statistically significant differences between Trast-treated tumours and recurrent tumours previously treated with Trast. ^##^Statistically significant differences between GSI + Trast and Trast alone in recurrent tumours.

Real-time RT–PCR was performed to detect canonical Notch target gene transcripts that include *HEY1* and *Deltex1* (Kopan and Ilagan, 2009) as measures of Notch signalling and efficacy of the LY 411 575 (Figure 3A) or MRK-003 GSI (Figure 3B) on the Notch pathway. Figure 3A and B demonstrate that LY 411 575 or MRK-003 GSI alone significantly inhibited *HEY1* and *Deltex1* transcripts compared with vehicle control. Trastuzumab treatment alone significantly increased *HEY1* by 2–4-fold and *Deltex1* by 20-fold compared with vehicle control (Figure 3A and B). LY 411 575 or MRK-003 GSI significantly decreased the trastuzumab-induced increase in *HEY1* and *Deltex1* (Figure 3A and B). In addition, recurrent tumours that grew post-trastuzumab treatment showed increased baseline expression of *HEY1* and *Deltex1* transcripts when compared with trastuzumab-treated tumours (Figure 3A). In contrast, recurrent tumours post-trastuzumab plus LY 411 575 GSI treatment showed decreased expression of *HEY1* and *Deltex1* transcripts compared with trastuzumab alone (Figure 3A). These results would indicate that the lack or delay of breast tumour recurrence observed for the combination of trastuzumab plus GSI treatment could be because of the prevention of the trastuzumab-induced increase in Notch signalling.

### Tumour histology, proliferative, and apoptotic characteristics of ErbB-2-positive breast tumour xenografts

**Figure 4:**
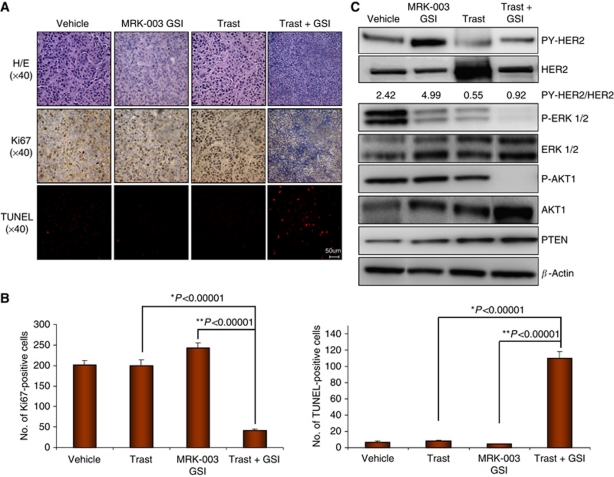
BT474 xenograft histology and signalling pathways. Five mice were euthanised and tumours excised at week 12 as shown in Figure 1B. One half of the tumours were immediately fixed in formalin and the remaining half snap-frozen in liquid nitrogen for future study. Fixed tumours were paraffin embedded and sectioned for H/E staining (upper panel), Ki67 (middle panel), and TUNEL (lower panel) assays. All sections were photographed at × 40 magnification using a light microscope. Four panels are shown: BT474 tumours treated with vehicle, trastuzumab (Trast), MRK-003 GSI, or Trast plus GSI. Shown are representative photographs based on at least three tumours. (**B**) Quantification of Ki67- and TUNEL-positive cells of three tumours using 60 high-powered fields (HPFs) × at 40 magnification. The *y* axis represents the number of Ki67- or TUNEL-positive cells for 60 HPFs. The bar graphs are mean±s.d.. ^*^Statistical significance between trastuzumab and trastuzumab plus MRK-003 GSI (Trast + MRK-003). ^**^Statistical significance between MRK-003 GSI and Trast + MRK-003 GSI. (**C**) Expression of ErbB-2 and downstream signalling pathways in BT474 tumours. Bits of tumours (1 mg) that were snap-frozen in liquid nitrogen were homogenised and lysed in RIPA buffer containing protease and phosphatase inhibitors. Cellular debris was removed by centrifugation at 1000 g for 5 min at 4 °C. Supernatants were collected and 25 *μ*g of total protein loaded onto a 7% SDS–PAGE gel followed by western blotting to detect tyrosine phosphorylated ErbB-2 or HER2 (PY1248-HER2), total HER2, P-ERK1/2, total ERK1/2, P-AKT1, total AKT1, PTEN, and actin proteins. The western blot shown is a representative of three independent tumour samples with similar results. Density of bands corresponding to PY-HER2 and total HER2 for each treatment groups was quantified using ImageJ program (NIH, Bethesda, MD, USA) and expressed as a PY-HER2/HER2 ratio of area of the peak.

Haematoxylin and eosin staining of tumours excised at week 12, which is prior to trastuzumab-induced regression from Figure 1B, showed that tumours treated with vehicle, trastuzumab, or MRK-003 GSI alone appeared similar in histology (Figure 4A, upper panel). However, tumours treated with trastuzumab plus MRK-003 GSI contained vast numbers of pyknotic nuclei, a possible indication of cell death (Figure 4A, upper panel). Ki67 staining of tumours was used to detect proliferation. The vehicle control, trastuzumab, or MRK-003 GSI-alone treatments had similar numbers of Ki67-positive nuclei (Figure 4A, middle panel) that were quantified using at least 3–5 tumours per group and 60 HPFs (Figure 4B). In contrast, Ki67 staining was almost undetectable in trastuzumab plus MRK-003 GSI-treated tumours (Figure 4A, middle panel, and 4B). Furthermore, apoptosis as measured by TUNEL assay showed a 40% increase in TUNEL-positive cells from trastuzumab plus MRK-003 GSI-treated tumours, consistent with nuclear pyknosis observed by H/E (Figure 4A, lower panel, and 4B). These results indicate that the lack of tumour recurrence from trastuzumab plus MRK-003 GSI treatment is probably because of simultaneous induction of apoptosis and downregulation of proliferation.

### Expression of activated ErbB-2, ERK1/2, and AKT1 proteins

The ERK and AKT pathway are activated downstream of overexpressed ErbB-2 (Yarden, 2001; Yarden and Sliwkowski, 2001). Thus, we examined the status of these pathways in treated tumours. BT474 tumours excised at week 12 from Figure 1B were lysed and analysed by western blotting to detect tyrosine-phosphorylated ErbB-2 (PY1248), total ErbB-2, phosphorylated ERK1/2, total ERK1/2, phosphorylated AKT1, total AKT1, and PTEN proteins. Figure 4C demonstrates that MRK-003 GSI treatment of BT474 tumours increased PY-ErbB-2 protein compared with vehicle control. Trastuzumab alone showed an 80% decrease in PY-ErbB-2 protein *vs* vehicle control (Figure 4C). Interestingly, PY-ErbB-2 protein was reduced 60% with trastuzumab plus MRK-003 GSI compared with vehicle control (Figure 4C). Furthermore, although either MRK-003 GSI or trastuzumab alone decreased P-ERK1/2 and P-AKT1 compared with vehicle, only trastuzumab plus MRK-003 GSI decreased both P-ERK1/2 and P-AKT1 to almost undetectable levels (Figure 4C). This decrease in P-AKT1 was associated with increased PTEN protein levels (Figure 4C). These results would suggest that downregulation of proliferation and induction of apoptosis by trastuzumab plus MRK-003 GSI could be due at least in part to synergistic or additive inhibition of ERK1/2 and AKT1 activities, which are critical signalling pathways for proliferation and antiapoptosis, respectively.

### Antitumour activity of lapatinib, MRK-003 GSI, or combination in ErbB-2-positive breast tumours *in vivo*

**Figure 5:**
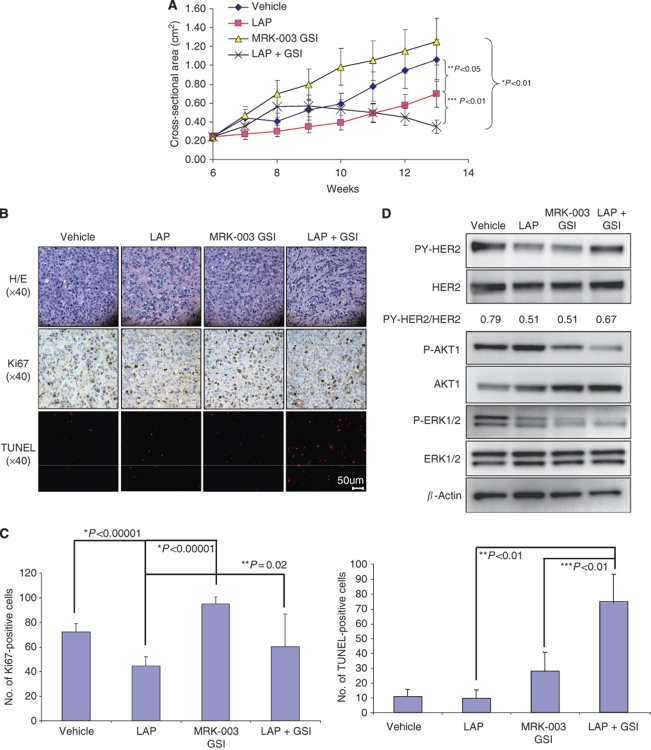
Lapatinib plus MRK-003 GSI reduces tumour growth. (**A**) ErbB-2-overexpressing BT474 xenografts were generated in 40 ovariectomised, athymic nude mice by injecting 5 × 10^6^ cells into both mammary fat pads. Once tumours reached a mean tumour cross-sectional area of 0.25 cm^2^, mice were randomised and treated with vehicle, lapatinib (LAP), MRK-003 GSI, or LAP plus GSI. Tumour area (length × width) was measured weekly using Vernier calipers. The measurements were performed up to 13 weeks. Results show mean tumour cross-sectional area ((area × Π)/4) on the *y* axis and time in weeks on the *x* axis. Error bars are s.d. of the mean for 10 mice bearing tumours. (**B**) Fixed tumours were paraffin embedded and sectioned for H/E staining (upper panel), Ki67 (middle panel), and TUNEL (lower panel) assays. All sections were photographed at × 40 using a light microscope. Four panels are shown: BT474 tumours treated with vehicle, lapatinib (LAP), MRK-003 GSI, and LAP + GSI. The photographs of a single tumour sample are representative of three tumours with similar results. (**C**) Quantification of Ki67- and TUNEL-positive cells of three tumours using 60 high-powered fields (HPFs) at × 40 magnification. The *y* axis represents the number of Ki67- or TUNEL-positive cells per 60 HPFs. The bar graphs are mean±s.d. ^*^Statistical differences compared with vehicle control. ^**^Statistical differences between LAP and LAP + GSI. ^***^Statistical difference between MRK-003 GSI and LAP + GSI. Statistical analysis was performed using a two-sided, nonpaired Student's *t*-test. (**D**) Bits of tumours (1 mg) that were snap-frozen in liquid nitrogen were homogenised and lysed in RIPA buffer containing protease and phosphatase inhibitors. Cellular debris was removed by centrifugation at 1000 g for 5 min at 4 °C. Supernatants were collected and 25 *μ*g of total protein loaded onto a 7% SDS–PAGE gel followed by western blotting to detect tyrosine phosphorylated ErbB-2 or HER2 (PY1248-HER2), total HER2, P-ERK1/2, total ERK1/2, P-AKT1, total AKT1, and actin proteins. Densitometry was performed on PY-HER2 and total HER2 bands for each treatment groups using ImageJ program and expressed as a PY-HER2/HER2 ratio of area of the peak. Western blotting was performed on at least three independent tumour samples. Representative western blots are shown with similar results. ^*^Statistically significant differences between mean slopes of the curve for LAP plus GSI and GSI alone. ^**^Statistically significant differences between mean slopes of the curve for LAP and vehicle control. ^***^Statistically significant differences between mean slopes of the curve for LAP plus GSI and LAP alone. Linear regression analyses were performed for tumour growth curve.

BT474 breast cancer cells contain ErbB-2 gene amplifications and are sensitive to lapatinib. Breast tumour xenografts were generated using BT474 lapatinib-sensitive cells in athymic nude mice. Growth of tumours was measured in response to vehicle, lapatinib, MRK-003 GSI, or lapatinib plus GSI. The results showed that lapatinib treatment decreased tumour growth by only 40% compared with the vehicle control (Figure 5A). Treatment of tumours using MRK-003 GSI alone had no statistically significant effect on tumour growth compared with the vehicle control (Figure 5A). However, lapatinib plus GSI showed significant reduction in tumour growth at week 13 compared with GSI alone, lapatinib alone, or vehicle (Figure 5A). The study was stopped at week 13 because of onset of diarrhoea in lapatinib-treated mice.

### Tumour histology, proliferative, and apoptotic characteristics of ErbB-2-positive breast tumour xenografts

Haematoxylin and eosin staining of tumours excised (Figure 5A) at week 10 showed that tumours treated with vehicle, lapatinib, MRK-003 GSI or lapatinib plus GSI appeared similar in histology and confirmed the presence of tumour in the samples excised (Figure 5B, upper panel). Ki67 staining of tumours was used to detect proliferation. The lapatinib, MRK-003 GSI, or lapatinib plus GSI treatments demonstrated 75–90% reduction in the Ki67-positive nuclei compared with the vehicle control (Figure 5B, middle panel, and 5C). Furthermore, apoptosis as measured by TUNEL assay showed a 10-fold increase in TUNEL-positive cells from lapatinib plus MRK-003 GSI-treated tumours compared with vehicle (Figure 5B, third panel, and 5C). These results indicate that induction of tumour regression from lapatinib plus MRK-003 GSI treatment is probably also because of simultaneous induction of apoptosis and downregulation of proliferation.

### Expression of activated ErbB-2, ERK1/2, and AKT1 proteins

BT474 tumours excised (Figure 5A) were lysed and protein extracts were analysed by western blotting to detect tyrosine phosphorylated ErbB-2 (PY1248), total ErbB-2, phosphorylated ERK1/2, total ERK1/2, phosphorylated AKT1 and total AKT1 proteins. Figure 5D demonstrates that lapatinib or MRK-003 GSI treatment alone decreased PY-ErbB-2 protein 40% compared with vehicle control. Interestingly, lapatinib plus GSI treatment, unlike trastuzumab, had little effect on PY-ErbB-2 protein. However, both phosphorylated ERK-1/2 and AKT1 proteins were reduced in tumours treated with lapatinib plus GSI compared with all other treatments (Figure 5D). These results indicate that induction of tumour regression by lapatinib plus MRK-003 GSI could also be because of simultaneous inhibition of both ERK1/2 and AKT1 activities.

### Growth of trastuzumab-resistant BT474 tumours *in vivo*

**Figure 6:**
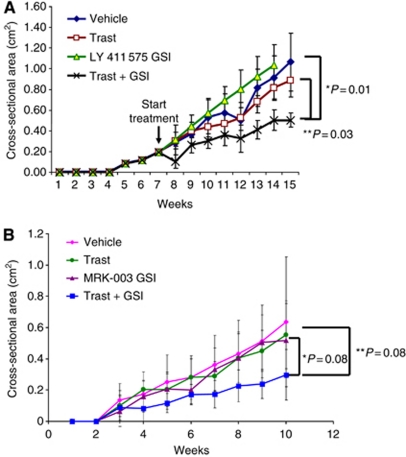
GSI partially restores trastuzumab sensitivity in resistant tumours. (**A** and **B**) The exact same protocol as described in Figure 1 was used to generate trastuzumab-resistant tumours in athymic, nude mice using BT474 trastuzumab-resistant cells. (**A**) LY 411 575 GSI was used. (**B**) MRK-003 GSI was used. Tumour area (length × width) was measured weekly using Vernier calipers. The measurements were performed up to 15 or 10 weeks, respectively. Results show mean tumour cross-sectional area ((area × Π)/4) on the *y* axis and time in weeks on the *x* axis. Error bars are s.d. of the mean for 10 mice bearing tumours. ^*^Statistically significant differences between GSI and Trast + GSI. ^**^Statistically significant differences between Trast and Trast + GSI.

Trastuzumab-resistant, ErbB-2-positive BT474 breast cancer cells were generated by treating cells with 10 *μ*g ml^−1^ trastuzumab for 6 months *in vitro* as described previously (Osipo *et al*, 2008). These resistant cells were injected into athymic, nude mice to generate trastuzumab-resistant breast tumour xenografts *in vivo*. Figure 6A and B show that xenograft tumours generated from trastuzumab-resistant cells were resistant to trastuzumab in two independent studies. Treatment with LY 411 575 (Figure 6A) or MRK-003 (Figure 6B) GSI alone did not inhibit tumour growth when compared with vehicle or trastuzumab alone (Figure 6A and B). However, trastuzumab plus LY 411 575 or MRK-003 GSI decreased the rate of tumour growth by almost 50% compared with GSI or trastuzumab alone (Figure 6A and B). Results from Figure 6A show significant reduction of tumour growth by trastuzumab plus LY 411 575 GSI. However, although results from Figure 6B demonstrate reduced tumour growth for trastuzumab plus MRK-003 GSI, this result did not reach statistical significance.

These data taken together suggest that the benefit of using a combination of trastuzumab plus GSI is prevention of tumour recurrence. However, once trastuzumab resistance occurs, a GSI can only partially restore trastuzumab sensitivity.

## Discussion

ErbB-2-positive breast cancer is currently treated with therapeutic agents trastuzumab (Carter *et al*, 1992) and lapatinib (Nahta *et al*, 2007), or current preclinical studies (Rimawi *et al*, 2011) and clinical trials are investigating the combination of trastuzumab plus lapatinib (Baselga *et al*, 2010). Although trastuzumab plus chemotherapy has been successful in the treatment of ErbB-2-positive breast cancer, some patients will not respond to this drug and, among responders, 15% will have disease recurrence and ultimately progression (Cobleigh *et al*, 1999; Vogel *et al*, 2002). Thus, trastuzumab resistance remains a serious clinical problem. One possible reason for this problem could be alterations in signalling pathways that are downstream or parallel to ErbB-2 upon trastuzumab or lapatinib treatment. We showed that ErbB-2 inhibition activates Notch-1 that results in a compensatory increase in Notch-1-mediated proliferation (Osipo *et al*, 2008). Moreover, high ErbB-2 expression in tumour-initiating cells of ErbB-2-positive breast cancer cell lines coincides with high Notch-1 expression and activity (Magnifico *et al*, 2009). Interestingly, increased ErbB-2 expression in tumour-initiating cells was shown to be Notch-1 dependent (Magnifico *et al*, 2009). Trastuzumab treatment was able to effectively target tumour-initiating cells of ErbB-2-positive breast cancer cell lines (Magnifico *et al*, 2009). However, Notch-1 has been implicated in not only the self-renewal of these tumour-initiating cells (Magnifico *et al*, 2009; Mine *et al*, 2009; Harrison *et al*, 2010), but also in trastuzumab resistance (Osipo *et al*, 2008; Huober *et al*, 2010). To circumvent these problems, we designed and evaluated for the first time a combination therapeutic strategy that can prevent and/or reverse trastuzumab resistance *in vivo.* Our data provide, to our knowledge, the first preclinical proof of concept in mice for future clinical trials of combination regimens including trastuzumab and a Notch inhibitor, MRK-003 GSI, for the prevention of tumour recurrence in ErbB-2-positive breast cancer.

Notch-1, a breast oncogene, is a modulator of cell-fate decisions (Politi *et al*, 2004). Overexpression of constitutively active forms of Notch-1, Notch-3, and Notch-4 develop spontaneous murine mammary tumours *in vivo* (Callahan and Raafat, 2001). Notch-4 has been shown to be critical for the survival of tumour-initiating cells (Magnifico *et al*, 2009; Mine *et al*, 2009; Harrison *et al*, 2010). In addition, Notch-1 has been recently suggested as a novel marker of trastuzumab resistance from human breast cancer tissue (Huober *et al*, 2010). We have identified Notch-1 as a novel target in trastuzumab-resistant breast cancer *in vitro* (Osipo *et al*, 2008). Our findings, along with evidence from the literature, indicate that Notch could be an important target in trastuzumab-resistant, ErbB-2-positive breast cancer. One class of compounds that are being used to inhibit the Notch pathway are GSIs that are currently in clinical trials for the treatment of breast cancer and other solid tumours (Pannuti *et al*, 2010). Recently, it was reported that MRK-003 GSI treatment of Balb/c-neuT female mice reduced tumour onset, tumour burden, and AKT1/mTOR activities associated with ErbB-2-positive, murine breast tumours (Efferson *et al*, 2010).

Our results demonstrate that although trastuzumab treatment caused virtually complete tumour regression (Figure 1A and B), which mimics what is observed in the clinic, it could not prevent tumour recurrence in 40% of the mice (Figure 1A and B, and 2). However, adding a GSI to trastuzumab treatment either completely abolished (MRK-003) or significantly reduced tumour recurrence (Figure 1A and B, and 2). Interestingly, recurrent tumours post-trastuzumab plus LY 411 575 GSI treatment showed a significant decrease in Notch transcriptional activity compared with trastuzumab treatment alone, suggesting that Notch signalling could be responsible for ErbB-2-positive breast tumour recurrence post-trastuzumab treatment. Consistent with the literature, our data could also suggest that Notch plays a critical role in the survival of tumour-initiating cells as demonstrated by the lack of tumour recurrence when Notch was inhibited in combination with ErbB-2 inhibition. Although the data presented here suggest a role for Notch based on Notch target genes modulated by a GSI, the effects on tumour recurrence could alternatively be the result of a *γ*-secretase-mediated effect as it is known that a GSI specifically inhibits *γ*-secretase activity.

Treatment with the combination of trastuzumab plus MRK-003 GSI simultaneously decreased proliferation and induced tumour cell death (Figure 4). These antitumour effects of the combination therapy may be because of near-complete blockade of two critical signalling pathways downstream of ErbB-2: ERK1/2 and AKT1 (Figure 4). Thus, a combination of trastuzumab plus MRK-003 GSI could benefit those women with recurrent, or possibly resistant, ErbB-2-positive breast cancer ultimately to reduce or eliminate disease progression and deaths by simultaneously inactivating two critical prosurvival and antiapoptotic pathways such as ERK1/2 and AKT1. The exact mechanism by which the combination treatment inhibits ERK1/2 and AKT1 activities is currently under investigation.

Lapatinib is a very potent inhibitor of ErbB-2 activity *in vitro*; however, lapatinib alone reduced tumour growth by only 40% in our BT474 model of ErbB-2-positive breast cancer (Figure 5A). A combination of lapatinib plus MRK-003 GSI showed significant reduction in the tumour growth (Figure 5A). This is likely because of inhibition of ERK1/2 and AKT1 activities (Figure 5D) that resulted in increased apoptosis and decreased proliferation (Figure 5B and C). However, the onset of diarrhoea-associated toxicity with lapatinib or lapatinib plus GSI treatment at week 13 caused the study to end prematurely and, therefore, complete tumour regression was not reached.

Our results also showed that a GSI could partially restore sensitivity to trastuzumab in resistant tumours (Figure 6). The mechanism by which a GSI only partially reverses trastuzumab resistance *in vivo* is being actively studied. It is possible that targeting all four Notch receptors with a pan-Notch inhibitor such as a GSI might not effectively target Notch-1, which we have shown to be necessary for trastuzumab resistance *in vitro* (Osipo *et al*, 2008). A more specific Notch-1 or possibly other Notch signalling pathway inhibitors could prove to be more effective and potent. We are currently investigating which components of Notch signalling should be targeted in trastuzumab- or lapatinib-resistant tumours to more effectively induce tumour regression. Therapeutic targeting of Notch receptors using antibodies could prove to be a more potent and specific mode of inhibition (Wu *et al*, 2010) and has not been fully investigated in breast cancer. Furthermore, the Notch pathway is complicated in tumours because of multiple modes of action. For example, it is known that Delta-like 4 on endothelial cells engages and activates the Notch-1 receptor on cancer epithelial cells to promote angiogenesis (Yan *et al*, 2010). In addition, Notch signalling has been recently implicated to play a role in survival and differentiation of tumour stroma (Orr *et al*, 2009). The level of complexity for the role of Notch signalling in the tumour microenvironment requires a thorough investigation of the Notch pathway in breast cancer, and most notably in anti-ErbB-2-targeted drug resistance, with the goal of identifying novel and specific targets to treat or reverse resistance.

We investigated the therapeutic benefits of two distinct inhibitors to block ErbB-2 (trastuzumab or lapatinib) and Notch (MRK-003 or LY 411 575 GSI) pathways. Although lapatinib is a more potent inhibitor of ErbB-2 activity *in vitro* compared with trastuzumab, trastuzumab is more efficient in inhibiting BT474 tumour growth. As the mice used in this study are immunodeficient, this is not likely to be an immune-mediated effect (e.g., ADCC). Lapatinib plus MRK-003 GSI were not as effective as trastuzumab plus MRK-003 GSI in inhibiting tumour growth of BT474 xenografts. A combination of MRK-003 GSI plus trastuzumab was sufficient to prevent tumour recurrence, whereas a combination of LY 411 575 plus trastuzumab was only able to reduce tumour recurrence. The observed differences in efficacy between MRK-003 and LY 411 575 GSI was also observed by recent data from Grudzien *et al* (2010), where the effect of MRK-003 was irreversible, leading to complete elimination of tumour-initiating cells *in vitro*, whereas the effect of LY 411 575 was reversible after drug washout. These results, in addition to data from the current paper, suggest that the best combination strategy for a future clinical trial to prevent tumour recurrence of ErbB-2-positive breast cancer could be trastuzumab plus MRK-003 GSI.

In conclusion, our findings suggest for the first time that the benefit of using a combination of trastuzumab plus a GSI is prevention of ErbB-2-positive breast tumour recurrence. Because Notch is a breast oncogene that is critical for survival and proliferation of breast cancer cells, our findings strongly suggest that combined treatment with a Notch inhibitor should be an effective therapeutic strategy to prevent tumour recurrence and possibly disease progression and death in ErbB-2-positive breast cancer. Our data also suggest that a combination of trastuzumab plus MRK-003 GSI could benefit women with recurrent, or possibly resistant, ErbB-2-positive breast cancer to prevent disease progression.
